# Cortical and trabecular bone are equally affected in rats with renal failure and secondary hyperparathyroidism

**DOI:** 10.1186/s12882-018-0822-8

**Published:** 2018-02-02

**Authors:** Nikita M. Bajwa, Cheryl P. Sanchez, Richard C. Lindsey, Heather Watt, Subburaman Mohan

**Affiliations:** 1 Musculoskeletal Disease Center, VA Loma Linda Healthcare System, 11201 Benton Street, Loma Linda, CA 92357 USA; 2 Department of Pediatrics, Loma Linda University, Loma Linda, CA 92354 USA; 3 Department of Medicine, Loma Linda University, Loma Linda, CA 92354 USA; 4 Department of Orthopedic Surgery, Loma Linda University, Loma Linda, CA 92354 USA

**Keywords:** Chronic kidney disease, Bone, Renal pathology, Phosphorus, FGF23

## Abstract

**Background:**

Changes in mineral metabolism and bone structure develop early in the course of chronic kidney disease and at end-stage are associated with increased risk of fragility fractures. The disruption of phosphorus homeostasis leads to secondary hyperparathyroidism, a common complication of chronic kidney disease. However, the molecular pathways by which high phosphorus influences bone metabolism in the early stages of the disease are not completely understood. We investigated the effects of a high phosphorus diet on bone and mineral metabolism using a 5/6 nephrectomy model of chronic kidney disease.

**Methods:**

Four-week old rats were randomly assigned into groups: 1) Control with standard diet, 2) Nephrectomy with standard rodent diet, and 3) Nephrectomy with high phosphorus diet. Rats underwent in vivo imaging at baseline, day 14, and day 28, followed by ex vivo imaging.

**Results:**

Cortical bone density at the femoral mid-diaphysis was reduced in nephrectomy-control and nephrectomy-high phosphorus compared to control rats. In contrast, trabecular bone mass was reduced at both the lumbar vertebrae and the femoral secondary spongiosa in nephrectomy-high phosphorus but not in nephrectomy-control. Reduced trabecular bone volume adjusted for tissue volume was caused by changes in trabecular number and separation at day 35. Histomorphometry revealed increased bone resorption in tibial secondary spongiosa in nephrectomy-control. High phosphorus diet-induced changes in bone microstructure were accompanied by increased serum parathyroid hormone and fibroblast growth factor 23 levels.

**Conclusion:**

Our study demonstrates that changes in mineral metabolism and hormonal dysfunction contribute to trabecular and cortical bone changes in this model of early chronic kidney disease.

## Background

The incidence and prevalence of chronic kidney disease (CKD) continues to rise, with rates doubling in people aged 65 years or older [1]. The pathological manifestations of the disease include renal hyperparathyroidism, cardiovascular disease, vascular calcification, and renal osteodystrophy, all of which can alter bone mineral metabolism resulting in increased fracture risk and other skeletal abnormalities [2–5]. Patients with moderate CKD can have up to a 2.4-fold increase in fracture occurrence compared to mild CKD or healthy patients [6].

CKD causes a dysregulation of mineral homeostasis that negatively alters bone mineralization parameters and leads to skeletal abnormalities [7, 8]. Several rodent models have been developed for CKD to evaluate the consequence and underlying mechanisms for bone metabolism changes in response to moderate CKD. In a 5/6-nephrectomy mouse model of CKD, the material properties of cortical bone within the tibial diaphysis were affected [9]. Similarly, a 5/6-nephrectomy rat model of CKD altered bone mineral and material properties and led to hyperparathyroidism [10]. In another model of CKD where rats underwent thyroparathyroidectomy and partial nephrectomy, cortical bone mineral properties in the femur were altered resulting in reduced mechanical strength [11]. In patients with CKD, common complications include hyperphosphatemia and secondary renal hyperparathyroidism (rHPT), in which disturbances in the homeostasis of calcium, phosphate, and vitamin D lead to elevated parathyroid hormone (PTH) levels and bone loss. Elevated PTH levels lead to reduced cortical [3] and trabecular bone mineral densities [12] as well as pronounced changes in trabecular microstructure [12–16].

In the absence of disease, calcium and phosphorus levels are maintained through a complex interaction between the kidneys, intestine, bones, and parathyroid glands. Calcium-sensing receptors in the parathyroid gland detect changes in serum calcium and regulate the synthesis and secretion of PTH [17]. Increases in PTH levels, as in CKD, occur as a response to increased renal phosphorus excretion and the downregulation of calcium sensing receptors that lead to sustained activation of PTH, thus causing detrimental effects on bone [18]. The disruption of phosphate homeostasis in CKD induces secondary hyperparathyroidism. Furthermore, higher levels of fibroblast growth factor 23 (FGF23), a bone-derived hormone that regulates phosphorous and 1,25[OH]_2_D3, may in fact precede changes in PTH levels [19]. Calcium, 1,25[OH]_2_D3, and PTH stimulate FGF23 secretion via negative endocrine feedback loops [20, 21]. Thus, PTH and FGF23 have been implicated as key players in mediating the observed changes in skeletal metabolism during kidney failure.

The effects of chronic renal failure on bone metabolism have been studied with nephrectomy animal models, and the 5/6 nephrectomy model is commonly used to induce severe secondary hyperparathyroidism. The aim of this study was to evaluate the effect of high phosphorus diet on the trabecular and cortical bone in rats with kidney failure and advanced secondary hyperparathyroidism using DXA scanning and micro-CT. We also aimed to determine the relationship between skeletal changes and serum levels of PTH, FGF23, and sclerostin.

## Methods

### Animals

Four-week old male Sprague-Dawley rats (Charles River, San Diego, CA) were used for this study. Animal housing and procedures were approved by the Institutional Animal Care and Use Committee of the Jerry L. Pettis Memorial Veterans Affairs Medical Center. Rats were anesthetized with isoflurane prior to surgical procedures. All procedures performed followed the EU Directive 2010/63/EU for animal experiments. At the completion of the study, rats were anesthetized with pentobarbital, followed by cardiac puncture until euthanasia.

**Fig. 1:**
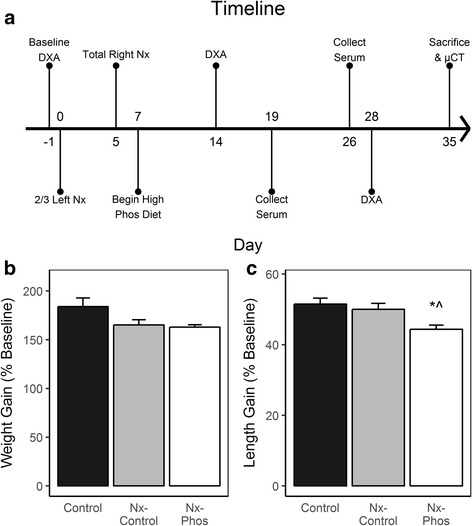
Study timeline and the effect of phosphorus on bone measurements. (**a**) Rats underwent baseline in vivo DXA imaging, followed by partial nephrectomy of the left kidney at day 0. At day 5, rats underwent total nephrectomy of the right kidney and began a high phosphorus diet or standard diet at day 7. In vivo DXA imaging occurred at 14 days post-nephrectomy, followed by serum collection 5 days later. One week later, serum was collected again, followed by in vivo DXA imaging at 28 days post-nephrectomy. At 35 days, rats were euthanized and bones underwent ex vivo micro-CT imaging. (**b**) Weight was monitored weekly throughout the duration of the study. Values are presented as mean percent increase over baseline ± SEM; *n* = 6–8. (**c**) Body length was obtained at baseline, prior to surgery and euthanasia. Values are presented as mean percent increase over baseline ± SEM; n = 6–8. *Significant at *p* < 0.05 versus Control animals. ^Significant at *p* < 0.05 versus Nx-Control animals

After an acclimation period of 1 week, rats were randomly assigned into the following 3 groups: 1) Control group fed with standard rodent diet (Control), 2) Nephrectomy group fed with standard rodent diet (Nx-Control), and 3) Nephrectomy group fed with high phosphorus diet (Nx-Phos). The standard rodent diet contained 0.68% phosphorus and 0.6% calcium. The high phosphorus diet used to induce advanced secondary hyperparathyroidism contained 1.2% phosphorus and 0.9% calcium. In order to maintain equal caloric intake between groups, the control group was pair fed to nephrectomized animals. Rats were maintained on these diets until completion of the study. Details of the experimental protocol are described in the text (Fig. 1a).

### Nephrectomy

Rats were deeply anesthetized and maintained at 1.5–2.5% isoflurane with oxygen. The 5/6 nephrectomy was performed as previously described [22]. Briefly, 2/3 of the left kidney was removed via a left paramedian incision on the back. The adrenal gland was carefully freed from the upper pole of the renal capsule before the renal pedicle was ligated and the kidney partially removed. Five days later, the right kidney was removed via a right paramedian incision on the back. For the control group, kidneys were manipulated without ablation. Rats were sacrificed under anesthesia 35 days after the first surgery. Kidneys from each animal were snap frozen in liquid nitrogen and stored at − 80 °C for protein extraction.

### Serum analysis

Blood was collected from rats on days 19 and 26 post-nephrectomy. Serum was stored at − 80 °C for future quantitation of intact FGF23 (EMD Millipore, Temecula, CA), phosphorus (Biovision Inc., Milpitas, CA), sclerostin (R&D Systems, Minneapolis, MN), calcium (Fisher Scientific, Hampton, NH), creatinine (Biovision Inc., Milpitas, CA), and intact PTH (iPTH) (Fisher Scientific, Hampton, NH) with ELISA kits. ELISAs were performed according to manufacturers’ recommended instructions.

### Evaluation of bone phenotypes

Cortical bone density and bone area of the total body, femur, tibia, and lumbar vertebrae (L4–6) were measured by dual-energy X-ray absorptiometry (DXA) using a PIXImus instrument (Lunar Corp) according to previously published methods [23, 24]. Trabecular and cortical bone parameters of the femur and L5 vertebrae were analyzed by microcomputed tomography (micro-CT; VIVA CT40, SCANO Medical) according to previously published methods [25, 26]. Micro-CT scanning was performed with 55–70 kVp (55 kVp for trabecular bone, 70 kVp for cortical bone) and a voxel size of 10.5 μm, and microarchitecture reconstructions were performed using the SCANCO software (SCANCO Medical).

### Histomorphometry

The tibiae were fixed and processed as previously reported [27]. Data were analyzed using the OsteoMeasure software (Osteometrics, Inc., Decatur, GA, USA), and measurements were taken at 10X magnification. Bone resorption in the secondary spongiosa of the distal tibia was measured with tartrate-resistant acid phosphatase (TRAP) staining. Oc.S/BS and Oc.Pm were measured according to established methods [28].

## Results

All rats survived to the completion of the study. There was no significant difference in body weight (Fig. 1b) between groups, but gain in total body length was reduced by 7.6% (*p* < 0.05) in the Nx-Phos vs. control and by 6.5% (*p* < 0.05) in the Nx-Phos vs. Nx-Control (Fig. 1c). See Table 1 for anthropometric measurements.

**Table 1:** Anthropometric measurements

Group	Initial Weight (g)	Final Weight (g)	Initial Body Length (cm)	Final Body Length (cm)	Final Femur Length (cm)
Control	95.77 ± 2.93	267.5 ± 4.0	25.09 ± 0.26	38.07 ± 0.03	3.164 ± 0.035
Neph-Control	97.60 ± 1.74	260.9 ± 4.7	25.51 ± 0.28	38.35 ± 0.09	3.151 ± 0.027
Neph-Phos	99.34 ± 1.30	257.6 ± 4.4	26.11 ± 0.23	38.18 ± 0.04	3.100 ± 0.025

Values are presented as means ± SEM

**Fig. 2:**
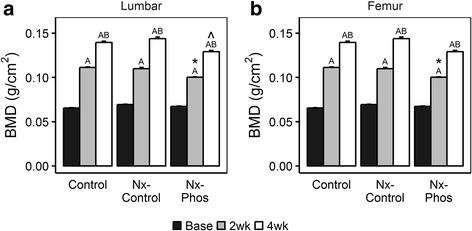
Effects of nephrectomy and/or high phosphorus diet on lumbar and femoral bone mineral density (BMD). (**a**) BMD was determined from DXA analysis of the lumbar vertebrae. ^A^Significant at *p* < 0.01 versus baseline rats. ^B^Significant at *p* < 0.01 versus 2 weeks post-nephrectomy. *Significant at *p* < 0.05 versus Control. ^Significant at *p* < 0.01 versus Nx-Control. Values are presented as means ± SEM; *n* = 11–14. (**b**) BMD was determined from DXA analysis of the femur. ^A^Significant at *p* < 0.01 versus baseline rats. ^B^Significant at *p* < 0.01 versus 2 weeks post-nephrectomy. *Significant at *p* < 0.05 versus Control. Values are presented as means ± SEM; n = 11–14

As expected, both femur and lumbar bone mineral density (BMD) increased across time in all 3 groups of rats. However, the lumbar BMD was significantly lower in the Nx-Phos compared to Nx-Control and control groups at 2 and 4 weeks post-surgery (*p* < 0.01; Fig. 2a). After 2 weeks, femur BMD was significantly reduced in the Nx-Phos group compared to the other groups (*p* < 0.05; Fig. 2b) but not at 4 weeks post-surgery

**Fig. 3:**
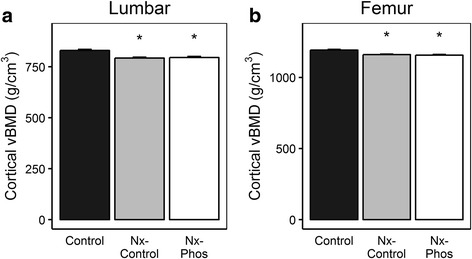
Effects of nephrectomy and/or high phosphorus diet on cortical bone. The (**a**) lumbar vertebrae and (**b**) femur vBMD were determined from micro-CT analysis. *Significant at *p* < 0.01 versus control. Values are presented as means ± SEM; *n* = 10–12

**Fig. 4:**
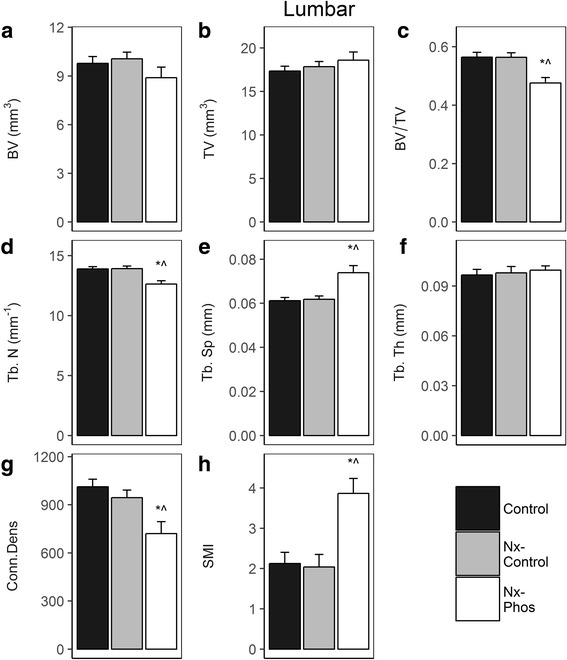
Effects of nephrectomy and/or high phosphorus diet on the microstructure of lumbar bone. (**a**) Bone volume, (**b**) total volume, (**c**) total volumetric BMD (BV/TV), (**d**) trabecular number, (**e**) trabecular separation, (**f**) trabecular thickness, (**g**) connectivity density, and (**h**) structure model index were determined from micro-CT analysis. *Significant at *p* < 0.01 versus Control. ^Significant at *p* < 0.01 versus Nx-Control. Values are presented as means ± SEM; n = 10–12

**Fig. 5:**
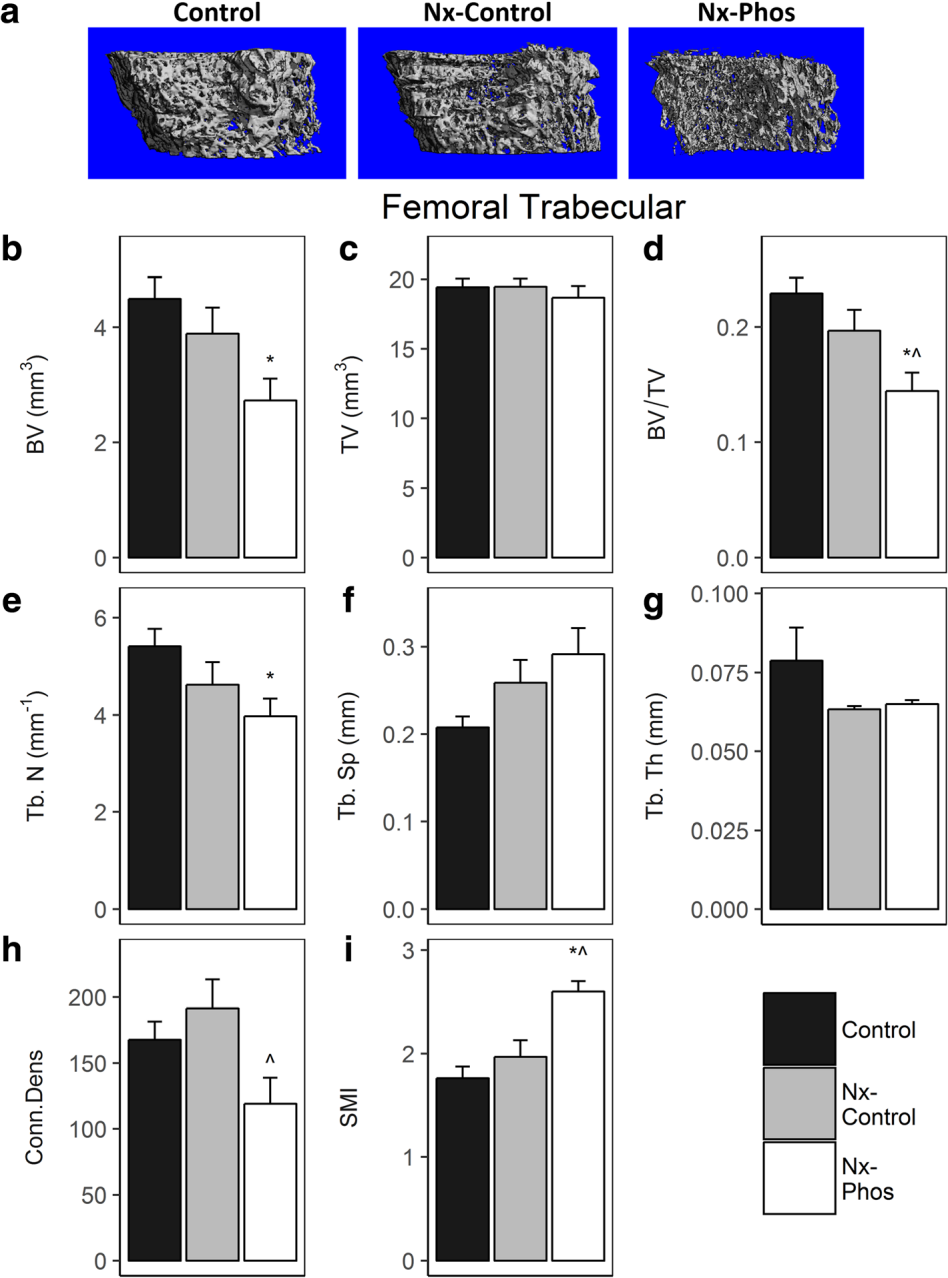
Trabecular bone parameters of the femur. (**a**) Representative micro-CT images, (**b**) bone volume, (**c**) total volume, (**d**) total volumetric BMD (BV/TV), (**e**) trabecular number, (**f**) trabecular separation, (**g**) trabecular thickness, (**h**) connectivity density, and (**i**) structure model index were obtained from micro-CT analysis. *Significant at *p* < 0.01 versus Control. ^Significant at *p* < 0.01 versus Nx-Control. Values are presented as means ± SEM; n = 10–12

Micro-CT analysis showed that nephrectomy triggered significant cortical bone loss. The volumetric bone mineral density (vBMD) in the fifth lumbar vertebrae was reduced by 4.5% in Nx-Control (*p* < 0.01) and 4.2% in Nx-Phos (*p* < 0.01) vs. Control (Fig. 3a). Femur cortical vBMD was also reduced by 2.6% in Nx-Control (*p* < 0.01) and 3% in Nx-Phos (*p* < .01) vs. Control group (Fig. 3b). Micro-CT analyses of trabecular bone of L5 vertebrae revealed that trabecular bone volume adjusted for tissue volume (BV/TV) was significantly reduced in Nx-Phos compared to Nx-Control and Control animals (*p* < 0.01; Fig. 4a–c). The reduced trabecular BV was caused by significant reductions in trabecular number (Fig. 4d) and increases in trabecular separation (Fig. 4e). In contrast, trabecular thickness was not different among the three groups of rats (Fig. 4f). The loss of trabecular elements reduced the number of interconnections between trabeculae as reflected by a > 25% reduction (*p* < 0.01) in connectivity density in the Nx-Phos compared to Nx-Control and Control groups (Fig. 4g). Accordingly, structure model index (SMI), which indicates relative prevalence of rod-like to plate-like structures, was increased in Nx-Phos animals (Fig. 4h). Similar to lumbar vertebrae, femurs of Nx-Phos animals exhibited significantly reduced trabecular BV that was caused by changes in trabecular number and thickness (Fig. 5a–g). Consistent with the changes in trabecular architecture, connectivity density was decreased while SMI was increased in the femurs of Nx-Phos animals (Fig. 5h–i). In contrast to bone volume (BV), measured tissue volume (TV) was not different between the three groups at the lumbar vertebrae and femurs (Fig. 4b and Fig. 5c).

Histomorphometric measurements at the secondary spongiosa of the tibia revealed increased bone resorption (TRAP-positive surface) caused by nephrectomy (Table 2). TRAP-positive osteoclast surface adjusted for bone surface (Oc.S/BS) was increased by 177% (*p* < .01) in Nx-C rats compared to Control. The Oc.S/BS was also increased by 72% (*p* < .05) in Nx-Control compared to Nx-Phos. Similarly, the osteoclast perimeter (Oc.Pm) was also increased in Nx-Control compared to Control rats, but did not reach statistical significance (*p* = .08).

**Table 2:** Histomorphometric measurements at the secondary spongiosa of the tibia

Group	Oc.Pm	Oc.S/BS
Control	0.63 ± 0.18	5.76 ± 1.63
Neph-Control	1.12 ± 0.19^#^	15.95 ± 2.86*
Neph-Phos	0.72 ± 0.21	9.25 ± 1.46^^@^

Values are presented as means ± SEM. Oc.Pm represents osteoclast perimeter; Oc.S/BS represents osteoclast surface adjusted for bone surface; ^#^*p* = .08 versus control; *p = 0.006 vs. control; ^*p* = 0.13 versus control; ^@^*p* = 0.04 versus Neph-Control

**Fig. 6:**
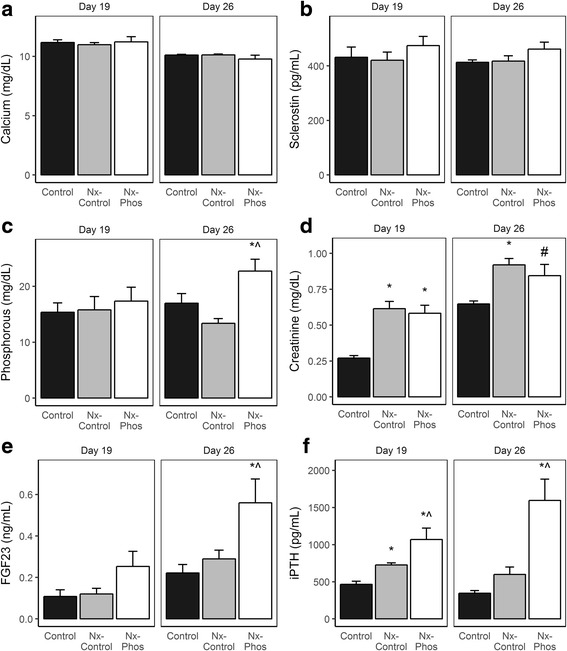
Effects of nephrectomy and/or high phosphorus diet on serum levels of systemic bone formation and skeletal markers. Serum levels of (**a**) Calcium, (**b**) sclerostin, (**c**) phosphorus, (**d**) creatinine, (**e**) FGF23, and (**f**) iPTH were measured at days 19 and 26 post-nephrectomy. *Significant at *p* < 0.01 versus Nx-Control. ^Significant at *p* < 0.01 versus Nx-Control. ^#^Significant at *p* < 0.06 versus Nx-Control. Values are presented as means ± SEM; *n* = 3–9

**Fig. 7:**
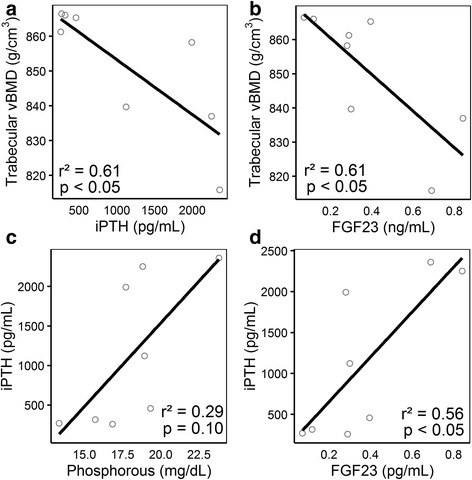
Relationships between bone and serum levels of systemic bone formation and skeletal markers in rats with nephrectomy and/or high phosphorus diet. (**a**) Trabecular volumetric bone mineral density (vBMD) vs. iPTH, (*r*^*2*^ = 0.61, *p* < 0.05), (**b**) trabecular vBMD vs. FGF23, (*r*^*2*^ = 0.61, *p* < 0.05), (**c**) phosphorus vs. iPTH, (*r*^*2*^ = 0.29, *p* = .10), and (**d**) iPTH vs. FGF23, (*r*^*2*^ = 0.56, *p* < 0.05) at day 26; *n* = 8

Serum levels of calcium (Fig. 6a) and sclerostin (Fig. 6b) were unaffected in all groups at days 19 and 26 after nephrectomy. Serum phosphorus (*p* < 0.01; Fig. 6c), creatinine (*p* = .05; Fig. 6d), and FGF23 (*p* < 0.01; Fig. 6e) levels were significantly elevated in Nx-Phos animals at day 26 post-surgery. In addition, nephrectomy induced a significant increase in serum creatinine and iPTH at day 19 post-surgery. The increases in serum PTH levels at day 26 were significant for the Nx-Phos but not for Nx-Control animals (*p* < 0.01; Fig. 6f). Serum levels of iPTH and FGF23 were negatively associated with trabecular vBMD (*r* = − 0.78, *r*^*2*^ = 0.61, *p* < 0.05; *r* = − 0.78, *r*^*2*^ = 0.61, *p* < .05; Fig. 7a & b, respectively) at day 26 after nephrectomy. Serum levels of phosphorus trended towards a positive association with iPTH (*r* = 0.54, *r*^*2*^ = 0.29, *p* = 0.10; Fig. 7c) at day 26 after nephrectomy. A positive relationship between iPTH and FGF23 (*r* = 0.75, *r*^*2*^ = 0.56, *p* < 0.05; Fig. 7d) was also observed at day 26 after nephrectomy. Interestingly, serum levels of sclerostin did not show significant correlation with bone parameters (trabecular or cortical bone), PTH, or FGF23 (data not shown).

## Discussion

Animal models of CKD use nephrectomy (partial or complete) as a method to induce early renal failure to accelerate endocrine dysfunction and bone turnover. Previous studies have shown the cortical region of bone to be the most susceptible to resorption in CKD [29, 30]. In this study, we investigated the effects of a high phosphorus diet on bone in a rat model of CKD and found that high phosphorus diet with advanced secondary hyperparathyroidism enhanced trabecular bone loss in the lumbar vertebrae and femur.

High levels of phosphorus have been found to increase vascular calcification, reduce cortical bone density and thickness, and cause severe trabecular bone alterations [2]. In a rat model of 5/6 nephrectomy, high phosphate reduced trabecular bone volume in the femur [31]. Similarly, we found that high phosphorus diet led to a significant change in bone microarchitecture as indicated by reduced trabecular bone volume, reduced trabecular number, and increased trabecular separation at day 35. These changes were also present in the lumbar vertebrae where the reductions in trabecular bone volume were caused primarily by increased trabecular separation. The changes in trabecular bone parameters cannot be explained based on systemic changes in somatic growth as neither body weight nor femur length were significantly different among the three groups.

To further explore the mechanisms behind trabecular bone loss, we examined the relationship between serum levels of PTH and skeletal alterations induced by high phosphorus diet. We found that high phosphorus diet increased PTH levels at days 19 and 26 and that increased PTH could have contributed to the increased bone resorption and reduced trabecular bone mass. PTH is known to enhance both bone formation and resorption [32]. In addition to exerting anabolic effects on osteoblasts, PTH treatment increases expression of receptor activator of nuclear factor kappa-B ligand, a key regulator of osteoclastic differentiation and activation [32]. PTH also promotes the conversion of 25-hydroxy vitamin D3, an inactive form of vitamin D3, into active 1,25-dihydroxy vitamin D3 (1,25[OH]_2_D3) by the activation of 1-α-hydroxylase in kidneys [33] leading to absorption of calcium and phosphorous in the gut. Several studies have investigated how PTH and 1,25[OH]_2_D affect serum phosphate via calcium regulation [34, 35]. Our data shows that nephrectomy alone increased serum PTH levels. However, this increase in PTH was further augmented by high phosphorus diet and confirmed by a positive correlative relationship. We also do report that serum levels of iPTH and FGF23 were negatively associated with trabecular bone volume; however, the issue of whether elevated PTH contributes to both cortical bone loss caused by nephrectomy and trabecular bone loss caused by high phosphorus diet in the nephrectomy rats remains to be evaluated. There are also data to suggest that there may be alternate mechanisms by which high phosphate affects trabecular bone, regardless of PTH levels [31].

To explore alternate mechanisms, we also examined the relationship between serum levels of FGF23 and skeletal parameters in the combined data from the 3 groups of animals. FGF23 has been reported to play a role in balancing mineral ion homeostasis and bone mineralization through the regulation of phosphorus [36, 37]. FGF23 is known to be secreted by osteocytes and regulate phosphorus homeostasis by reducing the reactivity of sodium-phosphate co-transporters in the proximal renal tubules and affecting 1,25[OH]_2_D metabolism and absorption by decreasing 1-α-hydroxyase activity [37–40], thus excreting excess phosphate through the kidney. In CKD and renal failure, FGF23 concentrations rise to excrete excess phosphate as a result of diminished renal function, leading to high bone turnover. We found that high phosphorus diet increased serum FGF23 levels at day 26. Additionally, high levels of serum FGF23 were strongly associated with trabecular bone loss. In our study, elevations in FGF23 were associated with increased phosphate and PTH levels, and all parameters were negatively associated with bone density, as other studies have reported [41, 42]. Sclerostin, like FGF23, is mainly secreted by osteocytes [43, 44]. Sclerostin decreases bone formation by inhibiting terminal differentiation of osteoblasts and promoting their apoptosis [45] in part via blockade of Wnt signaling pathways by binding to LRP5/6 receptors [45, 46]. In our studies, we did not find significant changes in serum levels of sclerostin in nephrectomy rats fed with normal or high phosphorus diet compared to control animals. However, we cannot exclude the possibility that local expression of sclerostin in osteocytes is altered by nephrectomy and/or high phosphorus diet. A recent study has also identified that FGF23/Klotho complex induces Dkk1 expression and thereby inactivates Wnt/β-catenin signaling in osteoblasts. Thus, impairment of Wnt/β-catenin signaling by high FGF23 levels may provide a mechanism for inhibition of bone formation in CKD [47]. In addition, the creatinine levels observed in our study are consistent with what others have reported in similar rat models of CKD [48–50].

Interestingly, nephrectomy induced significant cortical bone loss and higher osteoclast activity in the secondary spongiosa of the tibia, independent of phosphorus intake. Impaired kidney function may have altered the mineral or collagen composition of the cortical bone matrix [11, 51], which, in combination with PTH levels, may increase porosity [7]. In fact, higher PTH levels may predict cortical deterioration [3]. In addition, serum calcium levels were not reduced; this lack of an effect can be attributed to secondary hyperparathyroidism in nephrectomy rats.

### Limitations

First, this study utilized a hyperphosphatemia model that is not typically used since CKD patients would not be advised to increase phosphorus load. A low phosphorus diet has been found to suppress secondary hyperparathyroidism in mice with both mild and heavy proteinuria [52]. While we agree that it would be ideal to lower dietary phosphorus to prevent the development of hyperphosphatemia and the worsening of secondary hyperparathyroidism, patients, especially children with CKD, do not always follow low phosphorus intake recommendations due to food being unpalatable. Hence, the use of phosphate binding agents is frequently used. Our experimental design investigates the skeletal effects of hyperphosphatemia and severe secondary hyperparathyroidism in kidney disease. Dietary phosphorus restriction was not used in the Nx-Control group to compare the mild increase in PTH to severe secondary hyperparathyroidism. Second, the highly-scattered plots in Fig. 7 could be due to several reasons including the small number of replicates per group in the study, the biological variation in disease status in different animals, and the dilution of the relative contribution of measured parameters in CKD due to the involvement of multiple signaling pathways. Third, the cause and effect relationship between changes in PTH and FGF23 levels and trabecular bone microstructure in response to nephrectomy and high phosphorous diet remain to be investigated.

## Conclusion

Our study investigated the negative effects of renal failure and its relationship with phosphorus, FGF23, PTH, sclerostin, and bone. We found that high phosphorus diet adversely affects trabecular bone, whereas nephrectomy induced cortical bone loss. Future studies are needed to investigate the molecular mechanisms by which high phosphorus diet affects trabecular bone mass in models of CKD.

## Abbreviations

1,25[OH]_2_D3: 1,25-dihydroxy vitamin D3
BMD: Bone mineral density
BV: Bone volume
BV/TV: Bone volume adjusted for tissue volume
CKD: Chronic kidney disease
DXA: Dual-energy X-ray absorptiometry
FGF23: Fibroblast growth factor 23
iPTH: Intact parathyroid hormone
Oc.Pm: Osteoclast perimeter
Oc.S/BS: Osteoclast surface adjusted for bone surface
PTH: Parathyroid hormone
rHPT: Renal hyperparathyroidism
SMI: Structure model index
TRAP: Tartrate-resistant acid phosphatase
TV: Tissue volume
vBMD: Volumetric bone mineral density
Wnt: Wingless-type MMTV integration site family
